# Fifteen new nucleotide substitutions in variants of human papillomavirus 18 in Korea: Korean HPV18 variants and clinical manifestation

**DOI:** 10.1186/s12985-020-01337-7

**Published:** 2020-05-24

**Authors:** Namhee Kim, Jeong Su Park, Ji Eun Kim, Jae Hyeon Park, Hyunwoong Park, Eun Youn Roh, Jong Hyun Yoon, Sue Shin

**Affiliations:** 1 Department of Laboratory Medicine, Seoul National University Metropolitan Government Boramae Medical Center, Seoul, South Korea; 2 Department of Laboratory Medicine, Seoul National University College of Medicine, Seoul, South Korea; 3 Department of Laboratory Medicine, Seoul National University Bundang Hospital, Gyeonggi-do, South Korea; 4 Department of Pathology, Seoul National University Metropolitan Government Boramae Medical Center, Seoul, South Korea; 5 Department of Pathology, Seoul National University College of Medicine, Seoul, South Korea; 6 Department of Laboratory Medicine, Seoul National University Hospital, Seoul, South Korea

**Keywords:** Human papillomavirus (HPV) 18, *E6*, *E7* and *L1* genes, Variants, Lineage, Cervical cancer

## Abstract

High-risk human papillomavirus (HPV) infection is an essential factor for the development of cervical cancer. HPV18 is the second most common carcinogenic HPV type following HPV16, but the lineages of HPV18 have been less well studied than those of HPV 16. The purpose of this study was to analyze the nucleotide variants in the *E6*, *E7*, and *L1* genes of HPV18, to assess the prevalence of HPV18 variants in Korea and to explore the relationship between HPV18 genetic variants and the risk for cervical cancer.

A total of 170 DNA samples from HPV18-positive cervical specimens were collected from women admitted to a secondary referral hospital located in Seoul. Among them, the lineages of the 97 samples could be successfully determined by historical nomenclature.

All the studied HPV 18 variants were lineage A. Sublineages A1 and A4 comprised 91.7% (89/97) and 1.0% (1/97), respectively. Sublineages other than A1 or A4 comprised 7.2% (7/97). We identified 15 new nucleotide substitutions among 44 nucleotide substitutions: C158T, T317G, T443G, A560G, A5467G, A5560C, A5678C, A6155G, G6462A, T6650G, G6701A, T6809C, A6823G, T6941C and T6953C. Among them, 6 substitutions at positions 317, 443, 5467, 5560, 6462, and 6823 resulted in amino acid changes (E6: F71L and N113K; L1: H13R, H44P, A345T, and N465S, respectively). The pathologic results were classified as normal in 25.8% (25/97) of the women, atypical squamous cells of undermined significance (ASCUS) in 7.2% (7/97), cervical intraepithelial neoplasia (CIN) 1 in 36.1% (35/97), CIN2/3 in 19.6% (18/97), and carcinoma in 12.4% (12/97). There was no significant association between the HPV18 sublineages and the severity of pathologic lesion or the disease progression.

This study is the first to analyze the distribution of HPV18 variants in Korean and to associate the results with pathologic findings. Although the HPV18 variants had no significant effect on the degree and progression of the disease, the newly discovered nonsynonymous mutation in L1 might serve as a database to determine vaccine efficacy in Korean women.

## Introduction

Cervical cancer is the fourth most common cancer among all malignancies in females worldwide and the seventh most common cancer in Korea. According to the World Health Organization’s (WHO’s) GLOBOCAN project in 2018, 569,847 new cases occur and 311,365 people die annually due to cervical cancer worldwide. In Korea, cervical cancer is the seventh most common cancer, with the development of 3348 new cases and 1029 deaths reported annually [1]. Epidemiologic, genetic, immunological and environmental factors are involved in carcinogenesis, and persistent and high-risk human papillomavirus (HPV) infection is an essential factor for the development of cervical cancer. The most deleterious type is HPV16, and the second is HPV18; these two infections are associated with approximately 70% of cervical cancers [2, 3].

HPV is a small double-stranded DNA virus with an 8-kb genome containing early expressed genes (*E1*, *E2*, *E4*, *E5*, *E6*, and *E7*), late genes (*L1* and *L2*) and a long control region (*LCR*) [4]. The capsid proteins L1 and L2 play critical roles in viral structure formation and the infection process. In particular, purified L1, the major capsid protein, can form empty shells that resemble HPV, which are called virus-like particles (VLPs). These VLPs have hypervariable immunodominant loop structures on the surfaces of the virions that induce humoral immunity without oncogenic activity and are thus extensively used in HPV prophylactic vaccines [5–8]. *E6* and *E7* are major oncogenes that are highly expressed in tumors and are related to cellular immortalization, malignant transformation, and carcinogenesis. Based on these roles, proteins E6 and E7 are generally regarded as ideal targets for the development of therapeutic HPV vaccines [6, 9].

Over 200 HPV types have been identified based on *L1* sequences. HPV18 variants were originally grouped into European (E), Asian-Amerindian (AA) or African (AFR) lineages according to *E6-E7*, *L1*, and/or *LCR* sequences [10–14]. This classification has been superseded by a whole viral genome sequencing approach that has defined three major lineages (A, B, and C) and additional sublineages (A1 to A5 and B1 to B3) [15] that can be translated from the historical nomenclature (A1 and A2 are AA, A3 to A5 are E and B/C are AFR) [16, 17]. In addition, a recent study published in China proposed new A6 to A8 sublineages and classified them as the E lineage [18].

HPV genetic variants have been reported to differ in their viral assembly, pathogenic potential, and host immune response depending on geographic and ethnic features [2, 17–20]; however, no data on HPV18 variation among strains from Korean women have been reported thus far. Furthermore, there is some debate regarding the carcinogenic properties of HPV18 lineages [16–24].

## Methods

From 2010 to 2017, 7992 women admitted to the Seoul National University Boramae Medical Center were tested for cervical HPV genotype more than once. Among them, 3926 (3926/7992 = 49.1%) were positive for HPV, and 170 (170/3926 = 4.3%) were positive for type 18 and negative for other types. HPV detection and typing were performed using a liquid bead microarray, namely, the GeneFinder HPV PCR Kit (Infopia, Seoul, Korea).

Amplification and sequencing of HPV18 *E6*, *E7*, and *L1* genes were performed using type-specific primers, which are shown in Table 1 [11, 24, 25]. The cycling conditions were as follows: 5 min at 95 °C for initial denaturation; 45 s at 94 °C, 45 s at 55 °C, and 60 s at 72 °C for 35 cycles; and 10 min at 72 °C for final elongation. Amplicons were visualized on 2.0% agarose gels stained with ethidium bromide under UV transillumination. PCR products were automatically sequenced using the BigDye Terminator v3.1 Cycle Sequencing Kit (Applied Biosystems, Foster City, CA, USA) and an ABI 3730xl DNA analyzer (Applied Biosystems, Foster City, CA, USA) according to the manufacturer’s instructions. *E7* sequencing had a high success rate (165/170), so the sequencing was completed with only 1 primer set (=1 trial). However, *E6* and *L1* sequencing had low success rates, so we attempted 3 trials each (Table 1). All data were confirmed by repeating the PCR amplification and sequence analysis at least twice.

**Table 1:** Primers used for PCR amplification and sequence analysis

Gene	Primer	Trial	Primer sequence (5′-3′)	Position	Size	Success rate	Ref
*E6*	E6-a-F	1	AGAAACACACCACAATACTATGGCG	86	661	86/170	[21]
	E6-a-R		GTCGGGCTGGTAAATGTTGAT	746			
	E6-b-F	2	GGGACCGAAAACGGTGTAT	55	611	11/79	[22]
	E6-b-R		GAAGGTCAACCGGAATTTCA	665			
	E6-c-F	3	GGGACCGAAAACGGTGTAT	55	557	0/68	[22]
	E6-c-R		ATGTTGCCTTAGGCTCCATGC	611			
*E7*	E7-a-F	1	CGACAGGAACGACTCCAACGA	540	431	165/170	[21]
	E7-a-R		ATAAAACCAGCCGTTACAACCCGTG	970			
*L1*	L1–1-a-F	1	GTAACGGTCCCTTTAACCTCCTC	5402	650	153/170	[21]
	L1–1-a-R		CATTGTCCCTAACGTCCTCAG	6051			
	L1–2-a-F		AAGTTCCCATGCCGCCACGTCTAAT	6002	505	162/170	
	L1–2-a-R		AGAGCCACTTGGAGAGGGAGAATAC	6506			
	L1–3-a-F		GCTCTATTGTTACCTCTGACTCC	6502	636	32/170	
	L1–3-a-R		ATTACTTCCTGGCACGTACACGCAC	7137			
	L1–3-b-F	2	AGTTATGTATTTTGGGCTGTG	6077	756	0/128	[10]
	L1–3-b-R		ACACCAAAGTTCCAATCCTCTAA	6832			
	L1–3-b’-F		AGTATAGCAGACATGTTGAGGAA	6699	551	0/128	
	L1–3-b’-R		CATACAACATACAACAACAACCAT	7249			
	L1–3-c-F	3	TCCCTCTCCAAGTGGCTCTA	6488	601	44/128	[22]
	L1–3-c-R		AGTGGCAGATGGAGCAGAAC	7088			

Abbreviation: *Ref* reference

Nucleotide sequences were translated by the translate-tool of ExPASy (http://web.expasy.org/translate/) for the determination of amino acid changes. PSIPRED v.4.0 (http://bioinf.cs.ucl.ac.uk/psipred/) was used for secondary structure prediction, as it provides a simple and accurate secondary structure prediction method.

All sequence data were assembled against the HPV18 reference strain, GenBank sequence NC_001357, using Sequencher version 5.2.3 (Gene Codes Corporation, Ann Arbor, MI, USA). Twenty-eight other sequences were downloaded from GenBank, as in the latest Taizhou area study [18]. The accession numbers used in this analysis were as follows. A1 sublineage: EF202143-EF202145, A2 sublineage: EF202146, A3 sublineage: EF202147-EF202149, A4 sublineage: EF202150-EF202151, A5 sublineage: GQ180787, A6 sublineage: KY457833-KY457836, A7 sublineage: KY45737-KY45840, A8 sublineage: KY457826-KY457827; B1 sublineage: EF202153-EF202155, B2 sublineage: KC470224-KC470225, B3 sublineage: EF202152; and C lineage: KC470229-KC470230. Evolutionary analyses were conducted in MEGA X (http://megasoftware.net) [22, 23].

Based on cytological and histological evaluations of fresh specimens, the cervical lesions were graded according to their severity as follows: normal, atypical squamous cells of undetermined significance (ASCUS), low-grade squamous intraepithelial lesion (LSIL), high-grade squamous intraepithelial lesion (HSIL), cervical intraepithelial neoplasia grade 1, 2 or 3 (CIN1, 2 or 3) and cervical cancer. The histological diagnosis of each case was reviewed by an experienced pathologist who was unaware of the HPV testing results.

Mann-Whitney, Fisher exact and linear by linear association tests were used for comparisons between AA and E lineages. Variables affecting cervical cancer risk were analyzed by a logistic regression model. All statistical analyses were carried out with SPSS, version 22.0 (IBM, Armonk, NY, USA).

## Results

### HPV18 variants

Ninety-seven (97/170 = 57.1%) *E6*, 165 (165/170 = 97.1%) *E7*, 74 (74/170 = 43.5%) entire *L1* and 90 (90/170 = 52.9%) partial *L1* sequences from HPV18 isolates were successfully amplified by PCR and sequenced. In total, only 97 samples (*E6*-*E7*-*L1* sequences) could be classified as lineages and sublineages according to *E6*-*E7* sequence-based historical nomenclature rules of previous studies [9, 10].

**Fig. 1:**
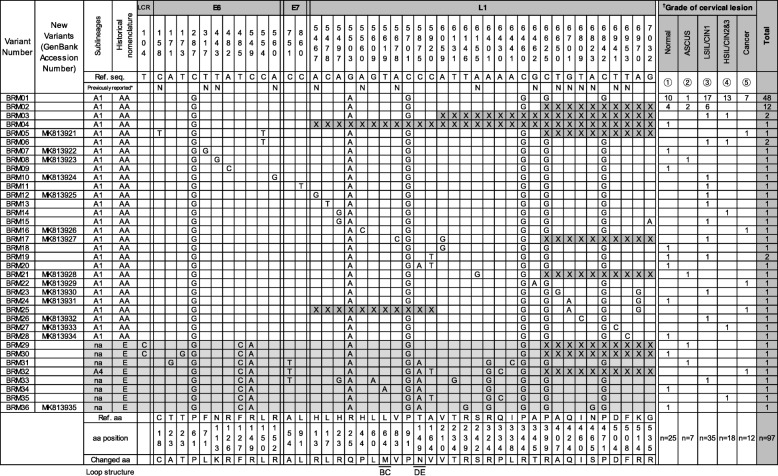
Nucleotide sequence variations in the HPV *E6-E7-L1* isolates in Korean women. ^*^Previously reported: N, No ^†^Grade of cervical lesions: ① Normal, ② ASCUS, ③ LSIL/CIN1, ④ HSIL/CIN2 and 3, ⑤ Cancer ^‡^L1 secondary structure: H, helix; C, coil; S, strand Abbreviations: AA, Asian-Amerindian; E, European; ASCUS, Atypical squamous cells of undetermined significance; LSIL, Low-grade squamous intraepithelial lesion; HSIL, High-grade squamous intraepithelial lesion; CIN, Cervical intraepithelial neoplasia; na, not assessed; aa, amino acid. *E6*, *E7* and *L1* nucleotide positions at which variations were observed are written vertically across the top. Amino acid translations and immunodominant loop structures of L1 are shown at the bottom. The GenBank accession numbers of the newly discovered sequences are on the left. The phylogenetic groupings based on the analysis of *E6*-*E7* are indicated. For each variant sequence, the positions that do not vary relative to the HPV reference are marked with a blank. The regions where sequencing failed are marked with an X. The cervical lesion grade for each variant is shown on the right

The genetic analysis of the regions of the 97 samples is shown in Fig. 1. They were composed of 26 intact and 10 partial *E6-E7-L1* sequences (BRM01 ~ 36 variants). In total, we found 44 nucleotide substitutions. Among them, 29 nucleotide substitutions were already reported: T104C, A171G, T173G, C287G, A482C, T485C, C549A, C554T, C751T, C860T, C5478T, A5497G, G5609A, T5619A, C5701G, C5875A, C5920T, A6059G, T6131G, T6146G, A6401G, A6430C, A6441C, C6460G, C6625G, C6842G, A6970G, and G7032A [11–14, 17, 18, 24, 26–29], and 15 nucleotide substitutions were newly identified: C158T of BRM05, T317G of BRM07, T443G of BRM08, A560G of BRM10, A5467G of BRM12, A5560C of BRM16, A5678C of BRM17, A6155G of BRM21, G6462A of BRM22, T6650G of BRM23, G6701A of BRM24, T6809C of BRM26, A6823G of BRM27, T6941C of BRM28, and T6953C of BRM36. Among the 15 new nucleotide substitutions, 6 nonsynonymous amino acid substitutions were at positions 317, 443, 5467, 5560, 6462, and 6823 (F71L and N113K in *E6*; H13R, H44P, A345T, and N465S in *L1*, respectively). In addition, all the 15 newly identified sequences in this study were submitted to GenBank under the accession numbers MK813921-MK813935 (Fig. 1).

**Fig. 2:**
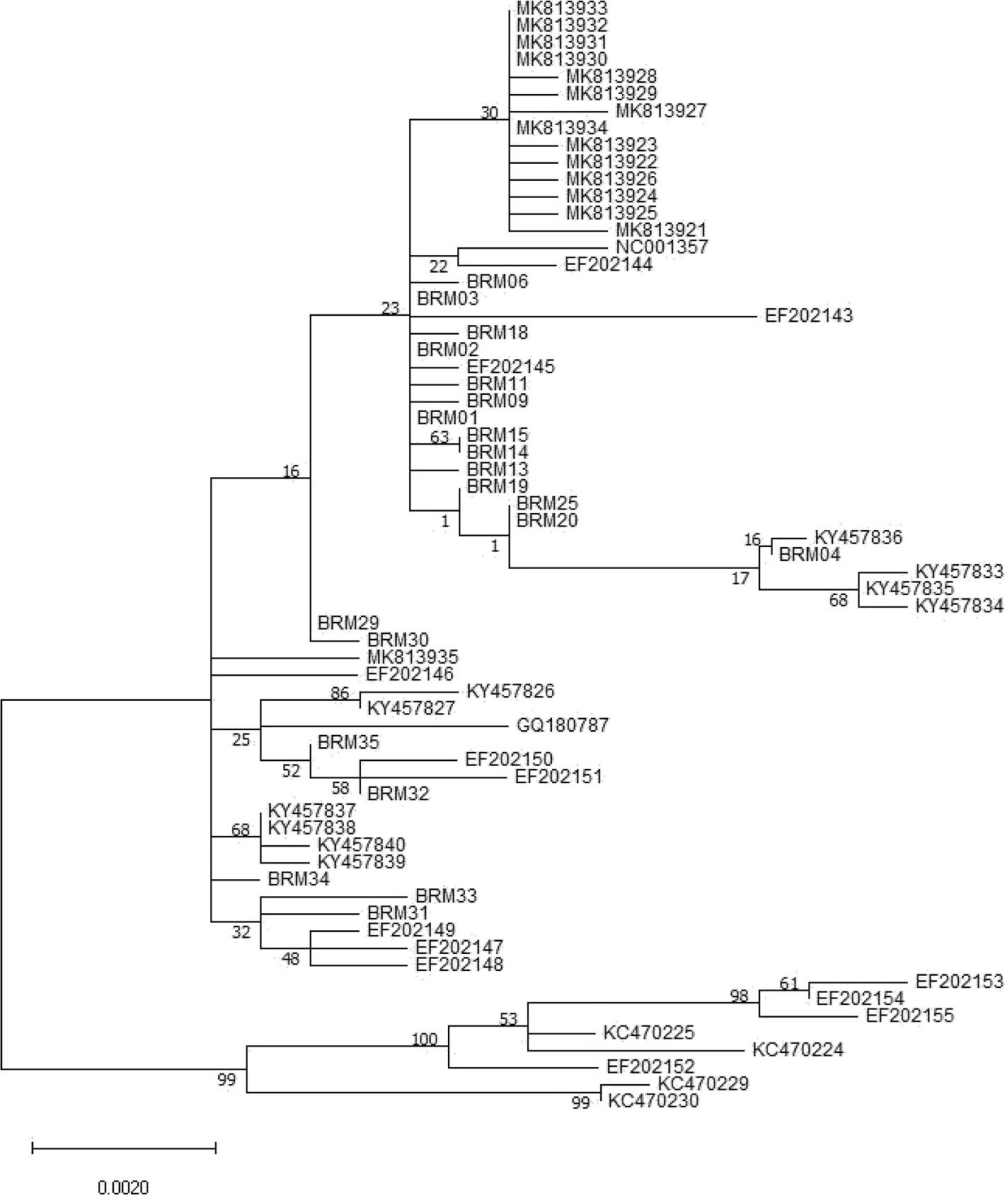
Phylogenetic tree of the HPV18 variants by the Maximum Likelihood method. The evolutionary history was inferred using the Maximum Likelihood method with 1000 bootstraps in a Tamura-Nei model. All positions with less than 95% site coverage were eliminated; i.e., fewer than 5% alignment gaps, missing data, and ambiguous bases were allowed at any position (partial deletion option). Numbers near the line indicate bootstrap values. Evolutionary analyses were conducted in MEGA X

The phylogenetic tree analysis was performed using the *E6-E7-L1* genes of the 97 Korean HPV18 isolates and 28 already reported variants (Fig. 2). According to the *E6*-*E7* sequence-based previous nomenclature rule [10, 11], all the previous AA lineages (BRM01 ~ 28, total 89 samples) were matched updated A1 sublineage sequences. However, except for one A4 sublineage (BRM32, 1 sample), the other seven variants (BRM29 ~ 31 and 33 ~ 36, total 8 samples) of the previous E lineage did not match the reported sublineages (Figs. 1, 2).

### Association between HPV18 variants and cervical lesion severity

All 97 of the women underwent pathological examinations, pap smears (cytological) and/or cervical biopsies (histological) after HPV genotyping within 3 months. The cervical pathologic results were classified as normal in 25 women (25/97 = 25.8%); ASCUS in 7 (7/97 = 7.2%); LSIL/CIN 1 in 35 (35/97 = 36.1%); HSIL/CIN2 and 3 in 18 (18/97 = 18.6%); and carcinoma in 12 (12/97 = 12.4%). There was no statistically significant association between HPV18 lineage and cervical lesion severity (Table 2).

**Table 2:** Distribution and statistical comparison of HPV18 lineages between the AA and E lineages

		AA(*n* = 89)	E(n = 8)	*P* value
**Age, years**		46 (40–54)	58 (47–63)	0.344^*^
**Cervical lesion, n**	Grouping			
Normal	①	22 (24.7%)	3 (37.5%)	0.298^†^
ASCUS	②	5 (5.6%)	2 (25.0%)	
LSIL/CIN1	③	34 (38.2%)	1 (12.5%)	
HSIL/CIN2&3	④	17 (19.1%)	1 (12.5%)	
Cancer	⑤	11 (12.4%)	1 (12.5%)	
**Pathology and treatment, n**				
Cancer	⑤	11 (12.4%)	1 (12.5%)	1.000^‡^
Non-cancer	① + ② + ③ + ④	78 (87.6%)	7 (87.5%)	
Surgery	④ + ⑤	28 (31.5%)	2 (25.0%)	1.000^‡^
Observation	① + ② + ③	61 (68.5%)	6 (75.0%)	
Definite dysplasia	③ + ④ + ⑤	62 (69.7%)	3 (37.5%)	0.110^‡^
Normal and ASCUS	① + ②	27 (30.3%)	5 (62.5%)	
**Evidence of co-infection, n**				
With other risk type(s)		21 (23.6%)	2 (25.0%)	1.000^‡^
With other high-risk type(s)		16 (18.0%)	1 (12.5%)	1.000^‡^
^**§**^**Serial follow-up testing (*n*** **= 54)**				
**Progression (n)**		*n* = 50	*n* = 4	
Progression		21 (42.0%)	1 (25.0%)	0.773^‡^
No progression		29 (58.0%)	3 (75.0%)	

^*^ Mann-Whitney test

^†^ Linear by linear association

^‡^ Fisher’s exact test

^§^ 54 out of 97 patients underwent serial pathological examinations

Grouping: ① Normal, ② ASCUS, ③ LSIL/CIN1, ④ HSIL/CIN2 and 3, ⑤ Cancer

Abbreviation: *AA* Asian-Amerindian; *E* European; *ASCUS* Atypical squamous cells of undetermined significance; *LSIL* Low-grade squamous intraepithelial lesion; *HSIL* High-grade squamous intraepithelial lesion; *CIN* Cervical intraepithelial neoplasia

Most women underwent HPV genotyping several times; 23 were found to have a different types of HPV infection, aside from type 18, in their lifetime. In particular, 17 women had an infectious history of other high-risk HPVs, including types 16, 31, 33, 35, 39, 45, 51, 52, 58, or 59, as diagnosed according to the International Agency for Research on Cancer (IARC) [30]. There was no association between HPV18 lineages and other types of HPV co-infection (Table 2). Additional infections by other types are not related to the development of cancer (data not shown).

Of the 97 women involved in this study, 54 women underwent an additional follow-up pathological evaluation. Twenty-two women had worsening lesions confirmed by serial pathological tests. There was no association between HPV18 lineages and disease progression (*P* = .773) (Table 2).

## Discussion

From 2010 to 2017, almost half (49.1%) of requested cervical HPV genotyping tests were positive for any type of HPV in a secondary referral hospital in Seoul, Korea. Among the positive results, the frequency of HPV 18 single positive was 4.2%. The frequency of the present study was comparable to other reports in Korea (total HPV prevalence was 16.7% ~ 40.7%, and the HPV 18 prevalence among HPV-positive women was 0.5% ~ 3.6%) [31–33].

The distributions of HPV variants differ among geographic origins, evolutionary dynamics, and pathogenicity. In our population, 6 substitutions, namely, C287G in *E6* and G5503A, C5701G, C6460G, C6625G, and C6842G in *L1*, were found in all HPV18 variants. These 6 substitutions were also found in all HPV18 isolates in southeastern and northeastern China [11, 18], which Korea is located next to, but found in 40% of HPV 18 in southwest and central China [24]. These findings support the geographical distribution of HPV lineages.

We previously discovered that the HPV 16 variants were composed of A1 ~ 3, A4, C, and D sublineages (54.1, 37.8, 0.7, and 7.4% of samples, respectively) in Korea [21]. Upon comparing HPV 16, little diversity was evident, indicating that previous lineages AA and E comprised 91.7 and 8.3%, respectively, in Korean HPV 18.

Previous studies reported that the risk of developing high-grade CIN is significantly increased with the non-European variants [13, 34]. One study [35] reported that the AA and European variants had significantly higher associations with pre-invasive lesions than the African variants. In contrast, other studies showed that no significant difference in pre-invasive lesion risk was observed between the variant lineages (A, B, and C) [14, 17, 26]. Our results are in line with the latter conclusion; there was no statistically significant association between HPV18 lineages and cervical pathologic lesions in Korea.

In the aspect of public health, many countries have implemented national policies of HPV vaccination. In Korea, HPV vaccination has been free for 12-year-old girls since 2016, and the government intends to expand the vaccination targets. Three prophylactic vaccines have received licensure from Korea Food and Drug Administration: the AS04-adjuvanted bivalent (HPV16/18) vaccine (Cervarix®, GlaxoSmithKline, Belgium), which was licensed in 2008; the aluminum hydroxyphosphate sulfate (AAHS) adjuvant quadrivalent (HPV6/11/16/18) vaccine (Gardasil®, Merck, US), which was licensed in 2007; and the AAHS adjuvant 9-valent (HPV6/11/16/18/31/33/45/52/58) vaccine (Gardasil®9, Merck, US), which was licensed in 2016. These prophylactic HPV vaccines are composed of L1 proteins of multiple HPV combinations [5, 7]. The loop structures of the HPV L1 major capsid protein contribute to the epitopes of vaccine-induced cross-neutralizing antibodies. Therefore, amino acid changes in the L1 loop region could be a critical issue for vaccination development. Our data on the genetic diversity of the HPV18 variants in Korea show two nonsynonymous substitutions in the loop structures, L64M within the BC loop (of BRM34) and T149N within the DE loop (of BRM20, BRM31 ~ 36). It may be helpful to design second-generation prophylactic HPV vaccines and implement feasible nationwide vaccination programs.

With the development of next-generation sequencing (NGS), the whole-genome sequencing (WGS) of 8 kb HPV became easier, making the analysis of lineages and single-nucleotide polymorphisms (SNPs) relatively faster and more accurate. However, WGS analysis pipelines in microbiological fields have not yet been established systematically, and it is difficult to analyze multiple samples with limited resources. In addition, although WGS identifies more variants and contributes to the construction of more accurate phylogenetic trees than partial sequencing, the data composed of *E6-E7-L1* sequences over the past 20 years does not have significantly reduced reliability compared to WGS. Thus far, many studies have been selectively conducted on the oncogenic proteins E6 and E7, and the major capsid protein L1 plays an important role in the prophylactic vaccine, as our study suggests.

## Conclusions

In summary, this research identified 15 new nucleotide substitutions and is the first to analyze the distribution of HPV18 variants in Korean women and to match the results to pathologic results. Data regarding the geographic/ethnic HPV18 genetic diversity distributions may be helpful for designing diagnostic probes, correlating epidemiologic cervical cancer risks, and analyzing the efficacy of HPV vaccines for targeted populations in Korea.

## Data Availability

The datasets used and/or analyzed during the current study are available from the corresponding author on reasonable request.

## Abbreviations

AA: Asian-Amerindian
AAHS: Aluminum hydroxyphosphate sulfate
AFR: African
ASCUS: Atypical squamous cells of undermined significance
CIN: Cervical intraepithelial neoplasia
E: European
*E*: Early gene
HPV: Human papillomavirus
HSIL: High-grade squamous intraepithelial lesion
IARC: International Agency for Research on Cancer
LSIL: Low-grade squamous intraepithelial lesion
*L*: Late gene
*LCR*: Long control region
NGS: Next-generation sequencing
SNP: Single-nucleotide polymorphism
VLP: Virus-like protein
WGS: Whole-genome sequencing
WHO: World Health Organization
